# Parafoveal OCT Angiography Features in Diabetic Patients without Clinical Diabetic Retinopathy: A Qualitative and Quantitative Analysis

**DOI:** 10.1155/2017/8676091

**Published:** 2017-06-29

**Authors:** Mathilde M. Goudot, Anne Sikorav, Oudy Semoun, Alexandra Miere, Camille Jung, Blandine Courbebaisse, Mayer Srour, Joseph G. Freiha, Eric H. Souied

**Affiliations:** ^1^Department of Ophthalmology, Centre Hospitalier Intercommunal de Creteil, University Paris Est Creteil, Creteil, France; ^2^Centre de Recherche Clinique and Centre de Ressources Biologiques, Centre Hospitalier Intercommunal de Creteil, Creteil, France; ^3^Department of Diabetology, Centre Hospitalier Intercommunal de Creteil, University Paris Est Creteil, Creteil, France; ^4^INOVAD Institution, Paris, France

## Abstract

**Purpose:**

To evaluate the capacity of OCT angiography (OCTA) for detecting infraclinical lesions in parafoveal capillaries in diabetic patients without diabetic retinopathy (DR).

**Methods:**

This prospective observational cross-sectional case-control study analyzed the superficial and deep capillary plexuses (SCP and DCP) on macular OCTA scans (3 × 3 mm) centered on the fovea. We compared 22 diabetic patients (34 eyes included) without DR diagnosis on color fundus photographs, with 22 age- and gender-matched nondiabetic controls (40 eyes included). Qualitative analysis concerned morphological ischemic capillary alterations. Quantitative analysis measured foveal avascular zone (FAZ) size, parafoveal capillary density, and enlargement coefficient of FAZ between SCP and DCP.

**Results:**

Neither the qualitative nor quantitative parameters were significantly different between both groups. No microaneurysms or venous tortuosity was observed in any of the analyzed images. On the SCP, the mean FAZ area was 0.322 ± 0.125 mm^2^ in diabetic patients and 0.285 ± 0.150 mm^2^ in controls, *P* = 0.31. On the DCP, the mean FAZ area was 0.444 ± 0.153 mm^2^ in cases and 0.398 ± 0.138 mm^2^ in controls, *P* = 0.20.

**Conclusion:**

OCTA did not detect infraclinical qualitative or quantitative differences in parafoveal capillaries of diabetic patients without DR in comparison with nondiabetic controls.

## 1. Introduction

Diabetic retinopathy (DR) is the leading cause of blindness in the working-age population in developed countries [1]. By 2035, estimates are that 592 million people will be affected by diabetes mellitus [2]. Early detection of its first signs plays a pivotal role in the management of DR, playing an important role in this significant public health issue [3]. Today, fundus slit lamp biomicroscopy and dilated 7-field stereoscopic 30° color fundus photographs are the current gold standard for DR screening (standard fields are described according to the Early Treatment Diabetic Retinopathy Study (ETDRS)) [4–6]. OCT angiography (OCTA) is a promising and simple test for retinal vascular examination. Nevertheless, to this date, there is a lack of evidence concerning its capacity to accurately analyze the retinal capillary network or its capacity to detect early DR changes, by comparison to standard screening tests.

OCTA is a recent noninvasive imaging technique that uses motion contrast to create retinal vascular networks images, by comparing the decorrelation signal among sequential OCT B-scans. In 2015, Spaide et al. demonstrated that OCTA could image separately each vascular layer of interest. The morphological retinal layer features in heathy eyes were described by Savastano et al. This type of specific analysis of either the superficial or the deep retinal capillary plexus or the choroidal vascular network is not possible with fundus fluorescence angiography [7, 8].

OCTA has previously been used to describe retinal microvascular lesions in diabetic patients and mainly macular ischemic lesions [9–22]. In diabetic patients without diabetic retinopathy, some studies have put forward a theory concerning infraclinical microvascular changes: the asymmetry and enlargement on the foveal avascular zone (FAZ) area [15, 16, 23, 24] and the presence of capillary nonperfusion areas adjacent to the FAZ [16, 25]. Because of its noninvasive nature and its rapidity, OCTA may become a good DR-screening tool. To date, only a few studies have evaluated OCTA in diabetic patients with no clinical DR, but no previous study has used color fundus photographs to ensure proper DR status classification (according to the Early Treatment Diabetic Retinopathy Study (ETDRS)) [26].

This study aimed to evaluate if OCTA can detect infraclinical lesions in the parafoveal capillary network of diabetic patients without diabetic retinopathy (DR) on color fundus photographs. The main outcome measure was the presence of significant differences in qualitative and/or quantitative OCTA features in the parafoveal superficial capillary plexus and/or the deep capillary plexus between diabetic patients without DR and age- and gender-matched nondiabetic controls.

## 2. Methods

In this descriptive prospective cross-sectional case-control study, 22 patients with type 1 or type 2 diabetes mellitus and with no clinical diabetic retinopathy (DR) nor diabetic maculopathy were examined. The study was conducted at the Department of Ophthalmology of the Creteil Hospital between March and September 2015. Twenty-two age- and gender-matched healthy control subjects, scheduled for regular visits, were also recruited during this time. Inclusion criteria were patients with type 1 or type 2 diabetes mellitus and the absence of diabetic retinopathy or maculopathy based on multimodal imaging performed on cases and controls by retina specialists (AS, AM, and MMG). The absence of clinical signs of DR was confirmed by two combined examinations: fundus slit lamp examination and counter analysis of 30° color fundus photographs. A macular spectral domain OCT scan (SD-OCT) established the absence of macular edema. The exclusion criteria were as follows: refractive errors over 6 diopters in spherical equivalent, severe media opacities, macular edema, and other retinal diseases like age-related macular degeneration, hypertensive retinopathy, central serous chorioretinopathy, and vitreoretinal interface, choroid, or optic nerve diseases. Each patient underwent a questionnaire concerning basic demographics, ophthalmological history, and cardiovascular risk factors. We collected the following data: diabetes duration, glycated hemoglobin values, and glomerular filtration rate if available within the last month preceding inclusion. When the results were not available, new blood tests were performed. All patients underwent fundus biomicroscopy with slit lamp and digital color fundus photographs. As the current literature recommendations for DR screening [6, 25, 27, 28], we performed two 30° photographs per eye under pupillary dilatation: a posterior pole (centered on the macula) and a nasal pole (centered on the optic disc), using the Canon CR-2 Plus Retinal Camera (Canon Company, London, UK). Standard SD-OCT and Enhanced Depth Imaging-OCT (EDI-OCT) were performed using the Spectralis OCT (Heidelberg Engineering, Heidelberg, Germany). We also registered automatic measurements of retinal foveal thickness as well as the manual measurements corresponding to subfoveal choroidal thickness. Choroidal thickness was measured on EDI-OCT scan at the center of the fovea from the posterior face of the retinal pigment epithelium to the choroidal/scleral junction. In accordance with the terms of the Declaration of Helsinki, the local ethics committee in Creteil approved the study. Informed consent was obtained from all subjects prior to the investigation-related procedures.

### 2.1. OCT Angiography (OCTA)

OCTA scans were acquired using a commercially available RTVue XR Avanti with the AngioVue OCTA software (Optovue Inc., Fremont, CA, USA). The scanning area was captured in 3 × 3 mm sections, automatically centered on the fovea. The split-spectrum amplitude decorrelation angiography (SSADA) algorithm [29] revealed the blood flow areas by the decorrelation of two cross-sectional 3D raster scans in order to improve the signal-to-noise ratio and minimize motion artifacts. Each 3-dimensional volume was obtained from 2 horizontal and 2 vertical fast B-scan acquisitions, composed of 216 B-scans and acquired in 3.4 seconds each. Each B-scan was composed of 304 × 304 A-scans acquired in a rate of 70,000 A-scans per second, using a scan beam centered on 840 nm, with a bandwidth of 45 nm. To improve the resolution of images, acquisitions could be repeated during the examination. Motion Correction Technology (MCT™) reduced motion artifacts, and the software ReVue reduced axial and transversal saccadic motion artifacts. To analyze the retinal vascular network, we used the automated segmentation of the superficial and the deep retinal capillary plexuses (SCP and DCP) included in the RTVue XR Avanti, as previously described [7, 22]. The preset settings were manually adjusted if automated segmentation errors (due to lack of fixation or cooperation) were present.

### 2.2. Qualitative and Quantitative Analysis

**Figure 1:**
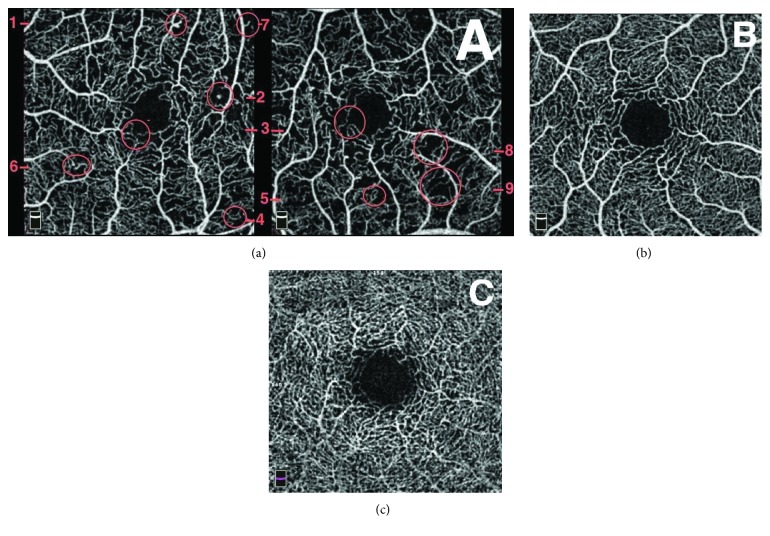
Reference OCT angiography (OCTA) scans for readers for the qualitative analysis of the capillary network. Typical abnormalities in a patient diagnosed with mild nonproliferative diabetic retinopathy on color fundus photographs (a): microaneurysms surrounded by capillary nonperfusion area (1, 2), punched out foveal avascular zone (FAZ) outline (3), capillary loops surrounded by capillary nonperfusion area (4, 5), blind-end capillaries (6, 7), and capillary nonperfusion area (3, 9). The images (b) and (c) are reference OCTA scans of a 24-year-old healthy control in the superficial capillary plexus (SCP) (b) and the deep capillary plexus (DCP) (c). In the SCP (b), the FAZ outline is clear and uninterrupted and the capillary plexus is regular, without nonperfusion area. In the DCP (c), the FAZ outline is less clear but still uniform and symmetrical, and no capillary loss appears.

Three retina specialists examined OCTA scans for the qualitative analysis of parafoveal microvasculature. All patients' and controls' 3 × 3 mm OCTA scans were therefore arranged on a slide show to enable a magnified analysis. Thesuccession of images was randomly determined in order to avoid letting the readers know the diabetic status of patients. Each OCTA scan was evaluated next to the corresponding “en face” acquisition in order to distinguish vitreous condensations, motion or position artifacts from capillary loss. We retained the following abnormalities, commonly concordant with DR in literature [11, 12, 14, 16–18, 24, 30, 31]: microaneurysms, venous tortuosity [16], irregular outline, and dropout of the anastomotic vascular arcade of the fovea avascular zone (FAZ) [18, 20, 21]. On the parafoveal capillary network, we noticed capillary loss and modified capillary shapes, that is, dilated capillaries, capillary loops [14, 32, 33], and blind-end capillaries [15]. Capillary loss were defined as nonperfusion areas characterized by a dark zone without any flow signal, with loss of speckled hyperreflectance homogeneity [21], surrounded by large retinal vessels [11, 34] and not compatible with artifacts. A microaneurysm was defined as a round, saccular, or fusiform focal hypersignal [17]. A blind-end capillary is a dilated interrupted terminal vessel surrounded by an area of capillary loss [15]. Typical images of these abnormalities and a normal OCTA scan of each plexus were given to readers as a reference before the analysis (Figure 1()).

**Figure 2:**
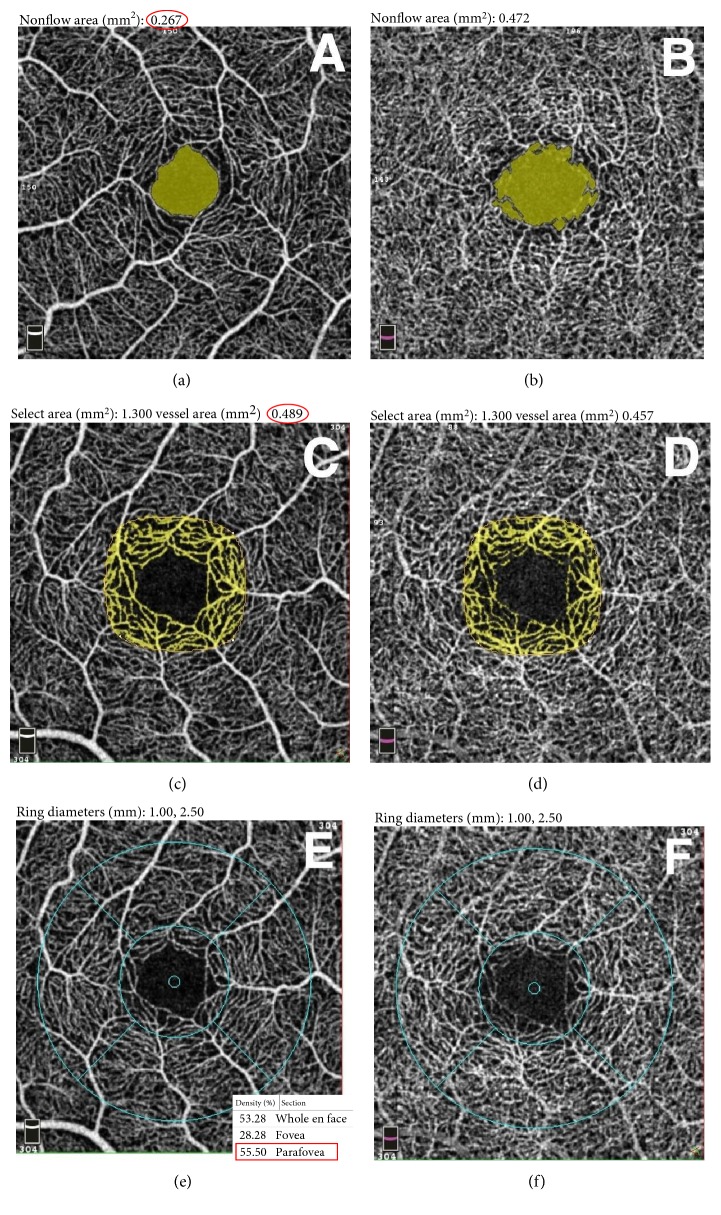
Quantitative analysis of the central retinal capillary network in the superficial capillary plexus (SCP) (left) and the deep capillary plexus (DCP) (right) on OCT angiography scans using the automatized tool of Angioanalytics software on RTVue. The foveal avascular zone area in SCP (a) and DCP (b) was measured using the “nonflow” detection tool. The examiner selected a point in the interest area and the software automatically coloured it in green and measured the area in mm^2^ (red circle). Parafoveal vascular density was evaluated with 2 different methods (c–f). In the first method, the “flow” detection tool measured the area of vessels in mm^2^ (yellow network, value circled in red) in a central region of interest manually determined at 1.3 mm^2^ in SCP (c) and DCP (d). In the second method, the “density” tool measured the vascular density in % in a 1 to 2.5 mm radius parafoveal ring in SCP (e) and DCP (f). The value framed in red is the mean density of the whole parafoveal ring noticed for our analysis.

Quantitative analysis was performed by one retina specialist (MMG) with Angioanalytics quantification software of Optovue system. We measured three vascular parameters: FAZ area, parafoveal vascular density, and parafoveal intercapillary area. The “nonflow area” tool automatically defines borders of FAZ and measures its area (Figures 2(a) and 2(b)). The reader could manually draw the boundaries in case of inaccuracies. The following parameters were calculated: the coefficient of enlargement of FAZ between the SCP and the DCP (resp., sFAZ and dFAZ) denoted *C*. This *C* value was calculated by the difference between the FAZ area of both plexuses normalized with sFAZ: *C* = (dFAZ − sFAZ)/sFAZ. The vascular density of the parafoveal area was assessed by 2 different methods. With the first method, the “flow area” tool quantified the area of vessels, denoted *A*, in a 1.3 mm^2^ user-defined region of interest, centered on the fovea (Figures 2(c) and 2(d)).

**Figure 3:**
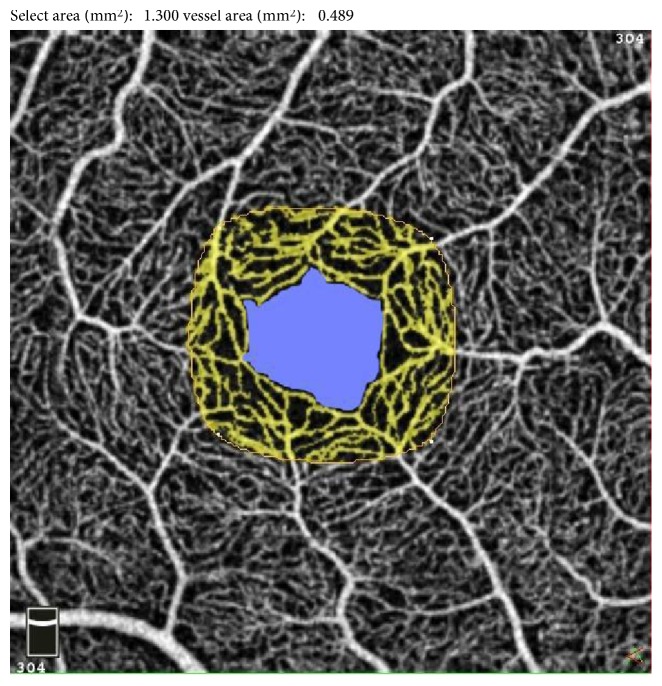
Representation of the first measurement's method of the parafoveal vascular density. The “flow” quantification tool measured the area of vessels in the central 1.3 mm^2^ user-defined region (yellow). The vascular density in % in the corresponding parafoveal ring (*D*) was then obtained with the formula *D* = *A*/(1.3 − FAZ) with FAZ (mm^2^) = area of the foveal avascular zone (blue) and *A* (mm^2^) = vessels' area in the central 1.3 mm^2^.

This selected area corresponded to the juxtafoveal region where capillary losses had been described in previous studies in patients without DR [16, 23]. The vascular density of the corresponding parafoveal ring, denoted *D*, was then obtained with the formula *D* = A/(1.3 − FAZ). In this formula, *D* = vascular density of the parafoveal ring in the central 1.3 mm^2^, FAZ = area of the foveal avascular zone in mm^2^, and *A* = capillary flow area of the central 1.3 mm^2^, as shown in Figure 3. With the second method, the “flow density map” tool assessed the relative density of vascular flow as a percentage of the total area: vascular density was measured in a 1 to 2.5 mm diameter parafoveal ring (Figures 2(e) and 2(f)). Parafoveal intercapillary area (PIA) of the central 1.3 mm^2^ was calculated by the difference between the parafoveal ring area and vascular area of the “flow” assessment tool with the formula PIA = 1.3 − FAZ − *A*, where FAZ = FAZ area (mm^2^) and *A* = vascular area of the central 1.3 mm^2^ (mm^2^).

### 2.3. Statistical Analysis

Qualitative variables are described in percentages. Comparisons of qualitative variables were performed using Mac Nemar's Chi^2^ test. The quantitative analysis compared each case to his age- and gender-matched control. Quantitative variables are described by their mean with standard deviation. Comparisons of means for paired variables were performed using the paired Student *t*-test. *P* values less than 0.05 were retained as significant. Analysis was performed with STATA version 13.0/SE (StataCorp. 2013. Stata Statistical Software: Release 13, College Station, TX, StataCorp LP). The coefficient regression values were calculated using the linear regression method. Multivariate analyses took into account all of the collected factors associated with the severity of the diabetic retinopathy [35]: age, duration of the diabetes, glycated hemoglobin level, and glomerular filtration rate calculated with the Modification of Diet in Renal Disease study equation (MDRD).

## 3. Results

We examined 22 diabetic patients (15 women and 7 men) without diabetic retinopathy and maculopathy at fundus slit-lamp examination, fundus photographs, and Spectral Domain OCT.  Table 1 summarizes the subjects' demographics and vascular risk factors. The mean age of patients was 51 ± 17 years, range (16–73). Thirty-two patients were Caucasian, 9 African, 1 Asian, and 2 Hispanic. The same multimodal imaging was performed with the 22 gender- and age-matched controls. All patients and controls underwent OCTA that includes a simultaneous acquisition of the corresponding OCT B-scan and OCT “en face.” In the analysis, we excluded fourteen eyes (10 diabetics and 4 controls) because of low-quality images. The final anonymized 74 OCTA scans of 34 diabetic and 40 control eyes were randomly disposed on a slide show in order to proceed to qualitative analysis (each slide contained one individual plexus scan). Readers looked for abnormalities blinded to patient status: diabetic or control.

**Figure 4:**
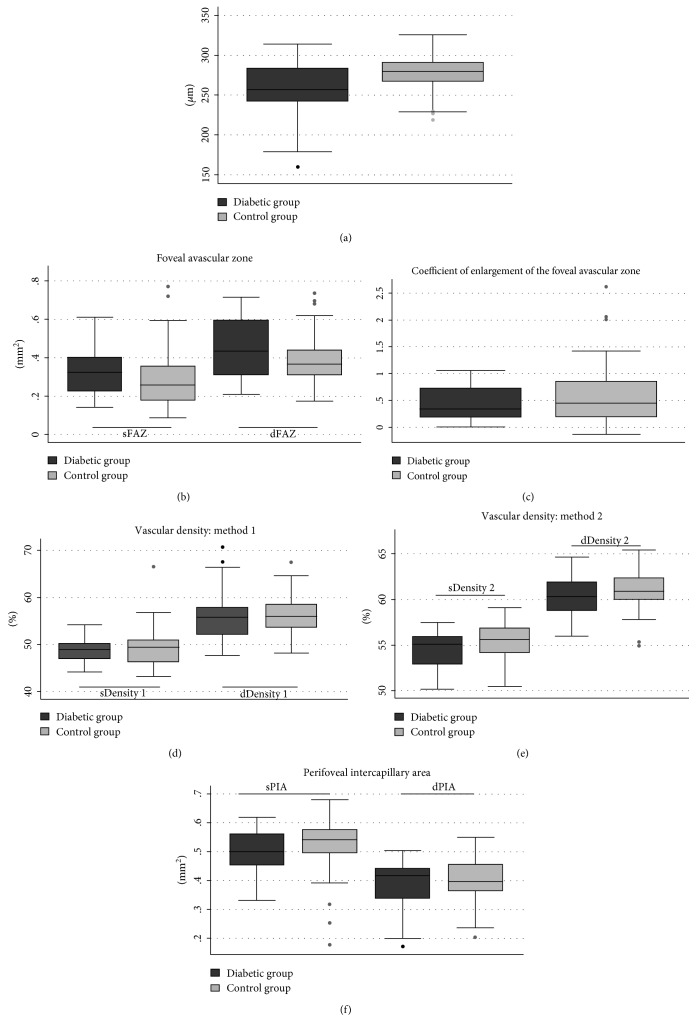
Comparison and scope of variation of the quantitative parameters of the parafoveal capillary network and the retinal foveal thickness in diabetic patients without DR and their age- and gender-matched controls. None of the quantitative parameters measured was significantly different between the two groups. The foveal avascular zone (FAZ) area in the superficial and the deep capillary plexuses (SCP and DCP) (a), the coefficient of enlargement between FAZ in SCP and DCP (b), the parafoveal vascular density measured with methods 1 (c) and 2 (d), and the parafoveal intercapillary area (PIA) (e) were comparable in both groups. The retinal foveal thickness was significantly thinner in diabetic patients, with a wide scope of variation (f).

Diabetic patients' clinical and laboratory findings are presented in Table 1. The diabetic patients' population consisted of 3 (14%) patients with type 1 diabetes and 19 (86%) patients with type 2 diabetes. Six (27%) patients had been referred at the first diagnosis of diabetes. Diabetic patients presented with an average glycated hemoglobin level of 10.5%, range (6.5–14.4). Seventeen (77%) patients took oral antidiabetes drugs and 9 (41%) patients used insulin. The mean duration of diabetes was 4.8 ± 4.1 years, range (0–13). The control of glycemia was variable, with a mean glycated hemoglobin level of 8.5 ± 2.9%, range (5.9–17). Besides diabetes, the diabetic group had a higher rate of additional cardiovascular risk factors than the control population, with a higher prevalence of hypertension (*P* = 0.05) and a higher body mass index (*P* < 0.001). There was no significant difference in other cardiovascular risk factors (tobacco consumption, *P* = 0.51; dyslipidemia, *P* = 0.25). Foveal retinal thickness was significantly thinner in diabetic patients (Table 2, Figure 4(a)): mean foveal retinal thickness was 257 ± 34 *μ*m in the diabetic group versus 277 ± 44 *μ*m in the control group, *P* < 0.01, CI 95% (−34; −7). Choroidal subfoveal thickness was not significantly different between both groups: 264 ± 64 *μ*m in cases versus 294 ± 68 *μ*m in controls, *P* = 0.06, CI 95% (−60; −1), and is concordant with the mean choroidal subfoveal thickness described in the current literature for healthy adult subjects [36].

In the qualitative analysis, no venous tortuosity and no microaneurysm was found in neither plexus for both groups. No difference was noticed either when looking for irregular outlines and punched out borders of the foveal avascular zone (FAZ), as well as nonperfusion areas or morphological capillary network abnormalities (i.e., dilated capillaries, capillary loops, and blind-end capillaries).

The quantitative analysis consisted in a paired Student *t*-test comparing each measured parameter of diabetic eye to his matched control eye. As presented in Table 2, none of the studied vascular parameters was significantly altered in the diabetic group without diabetic retinopathy (DR) by comparison to controls (Figure 4). On both plexuses, the FAZ area was not significantly different between the two groups but its mean was higher in diabetic patients (Figure 4(b)). The superficial FAZ (sFAZ) area measured 0.322 ± 0.125 mm^2^ in diabetic patients versus 0.285 ± 0.150 mm^2^ in controls, with a mean difference of 0.037 ± 0.223 mm^2^, CI 95% (−0.036; 0.109), *P* = 0.31. The deep FAZ (dFAZ) area measured 0.444 ± 0.153 versus 0.398 ± 0.138 mm^2^ with a mean difference of 0.046 ± 0.221 mm^2^, CI 95% (−0.025; 0.118), *P* = 0.20. There was no association between the areas of sFAZ or dFAZ and the age, the glomerular filtration rate, the duration, and the control of diabetes on univariate and multivariate regression analyses. The coefficient of enlargement of FAZ was not significantly different between both groups (Figure 4(c)). The mean coefficient of enlargement was higher in the control group, it was 0.43 ± 0.32 in cases and 0.60 ± 0.61 in controls, *P* = 0.14, CI 95% (−0.39; 0.06). Parafoveal vascular densities were not significantly different between the two groups in both measuring methods. Their means were lower in the diabetic group except for the parafoveal density in the deep capillary plexus measured with the first method by comparison to controls (Table 2, Figures 4(d) and 4(e)). The “flow” quantification (method 1) measured a lower density than the automatized measurement (method 2): for example, in the superficial capillary plexus with both groups values, the mean density was 49.2 ± 3.6% in the diabetic group and 55.1 ± 2.0% in controls, *P* < 0.001. The parafoveal intercapillary area of the central 1.3 mm^2^ was not significantly different between the two groups in both plexuses, and its mean was higher in diabetic patients (Table 2, Figure 4(f)).

## 4. Discussion

In the current global context of alarmingly escalating rates in prevalence and incidence of diabetes mellitus [3], efforts should be aimed towards understanding early retinal vascular changes in order to detect early biomarkers. This would allow for more efficient screening of diabetic retinopathy (DR), before complications arise.

The description of DR with OCT angiography is recent, and data in diabetic patients without clinical DR is scarce and not always concordant.

To our knowledge, this is the first study on OCTA using color fundus photographs to ensure proper DR status classification. The ETDRS classification has been established as a reference by comparing FA findings to fundus photographs [26], and studies have subsequently demonstrated better performance of mydriatic color fundus photographs by comparison to the fundus biomicroscopy for the screening of DR [5, 6, 37]. Furthermore, ophthalmologists often underscore DR in case of mild nonproliferative DR with few microaneurysms and microhemorrhages [5, 37].

**Figure 5:**
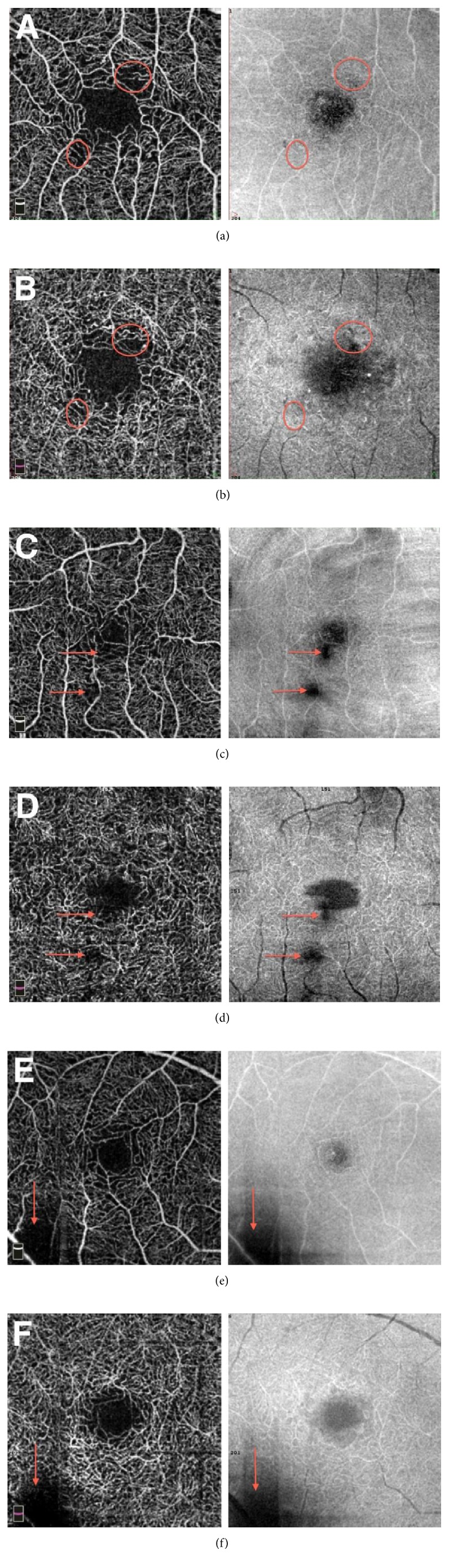
Interest of the “en face” OCT acquisition next to the 3 × 3 mm OCT angiography scan for the qualitative analysis to distinguish frequent focal artifactual losses of signal from real capillary nonperfusion areas. Acquisitions are presented successively in the superficial capillary plexus (SCP) (a, c, e) and the deep capillary plexus (DCP) (b, d, f). Real capillary nonperfusion area (a, b) are well marked out with clearly defined peripheral vessels and the surface of the signal's loss is different in both plexuses (red circles) (b). No loss of signal is noticed on the corresponding OCT “en face” acquisition (red circles). We distinguish those from vitreous condensations (c, d), which are responsible for a mask effect on all underlying layers (red arrows). Their borders are hazy, with peripheral blurred vessels and the lack of signal also present with an analogous shape on the “en face” acquisition (d). Position artifacts (e, f) are often located in corners and are also responsible for identical dark areas on both layers and on the “en face” acquisition (red arrows).

We previously defined a consensual description of the researched lesions on OCTA and transmitted images of typical abnormalities as references for readers (Figure 1). As we looked for very slight modifications of the microvasculature, the use of a software correcting artifacts, as well as the definition of relevant criteria for the detection of artifacts, was essential. Indeed, we noticed that the display of the “en face” acquisition was necessary to analyze OCTA scans. This was in order to prevent bias due to the fact that some artifacts resemble capillary loss (Figures 5(a) and 5(b)). Vitreous condensations (Figures 5(c) and 5(d)) and position artifacts (Figures 5(e) and 5(f)) are typical examples of flow signal deletion in the “en face” OCT and in all underlying vascular layers.

Qualitative descriptions of the parafoveal network in diabetic patients without DR in the literature show conflicting results. Some authors describe a clear and regular appearance of the foveal avascular zone (FAZ) borders of the superficial capillary plexus (SCP) as well as uniform perivascular spaces [15]. However, more recent studies describe significant alterations of the SCP in these patients. These alterations are defined with a composite criterion, which includes FAZ border and parafoveal capillary characteristics [16]. Some studies on healthy subjects have pointed that out.

FAZ outlines can often be irregular in the absence of diabetes [38]. Our qualitative analysis did not find any significant difference between both groups, considering irregular and punched out borders of the FAZ, nonperfusion areas, and morphological changes of the capillary network. No microaneurysm or venous tortuosity was found in OCTA scans in our included diabetic population.

Though enlargement of FAZ in DR [13, 15, 16, 18, 23, 24] has been well documented, data concerning quantitative capillary analyses are still not concordant in diabetic patients without DR [14–16, 23, 24] compared to nondiabetic controls (Table 3). Fundus fluorescence angiography did not find a significant enlargement of the sFAZ but measured a significant enlargement of the parafoveal intercapillary area in the SCP (*P* < 0.01, *N* = 7) [24]. In another study, OCTA observed a significant enlargement of FAZ in both superficial and deep capillary plexus (resp., in literature: *P* = 0.04, *N* = 53 and *P* < 0.01, *N* = 24) [15, 23]. However, this quantitative difference in FAZ area did not correspond to morphological changes of the regularity and appearance of FAZ in the concomitant qualitative analysis [15]. Our results did not show any significant enlargement of the FAZ in the superficial or the deep capillary plexus (sFAZ and dFAZ) of diabetic patients without clinical DR, by comparison to healthy controls.

As previous authors suggested predominant and earlier enlargement of FAZ in the deep capillary plexus (DCP) [14] in DR, we decided to look at this hypothesis: was the absolute value of FAZ area a good criterion to detect early DR? Because of its interindividual critical variability in healthy subjects [18, 20, 30, 38–43], we hypothesized that the naive FAZ area could be a confusing factor in FAZ enlargement's measurement. As a consequence, the coefficient of enlargement of the FAZ between the SCP and DCP was established to detect earlier deep vascular changes independently of individual FAZ variations. dFAZ is normalized with sFAZ so that each patient becomes its own control. This coefficient was not significantly higher in the diabetic group than in healthy controls (*P* = 0.14). Moreover, its mean value was higher in the control group with a mean of 0.60 ± 0.61 in cases and 0.43 ± 0.32 in controls. These results argue against the earlier alteration of deep vascular network in diabetic patients without DR. We noticed that this value was relevant only if sFAZ is not altered, as we observed it in our qualitative analysis.

The development of DR can be seen either in a linear progression or in a stairstep model. This study could lead to hypothesize a stairstep model of the apparition of ischemic lesions on OCTA, concordant with the first microhemorrhages and microaneurysms on color fundus photographs. The literature also describes obvious vascular lesions with OCTA in mild diabetic retinopathy [14, 15, 17, 18] arguing in favor of the high sensitivity of OCTA to detect early signs of clinical DR.

The interpretation of our results should take into account that the area of the retina analyzed with 3 × 3 mm OCTA scans is limited and that the precise correlation of macular ischemia and peripheral DR manifestations remains to be established. Further studies using wider field OCTA, with peripheral fundus photographs are necessary to evaluate this correlation. In our study, a single examiner (MMG) performed FAZ measurements using an automatic program imbedded in the software of the OCTA. Literature argues in favor of the reproducibility and repeatability of FAZ measurement with the same method [44]. The mean sFAZ and dFAZ area measured matched with the values of the literature (Table 3).

A noticeable characteristic of our patients is the short duration of diabetes (4.8 ± 4.0 years), with 6 patients examined at the discovery of the disease. Though it allows us to study the very beginning of the disease, we cannot exclude that capillary microchanges existing before clinical DR could not be described because of this relative short duration of diabetes.

In conclusion, OCTA did not detect infraclinical quantitative or qualitative differences in parafoveal capillaries of diabetic patients without DR in comparison with controls. This result suggests that color fundus photographs and OCTA could concord to assess the absence of DR. The increasing distribution of OCTA in the clinical ophthalmological practice and the development of full-field OCTA fundus camera should be able to strengthen these findings on a wider scale. Considering that it is relatively easy to perform, inexpensive, and noninvasive, OCTA could become an efficient screening tool and might create a breakthrough in the solving of one of the major eye health problems in developed countries.

## Figures and Tables

**Table 1:** Demographics, vascular risk factors, and characteristics of the diabetes.

		Diabetic group *N* = 22	Control group *N* = 22	*P* ^∗^
		Mean ± SD (range)		
Vascular risk factors	Age: years (matched)	51 ± 17 (16–73)	51 ± 17 (16–73)	0.24
	Women/men (matched)	7/15	7/15	—
	Hypertension: *N*	20	12	0.05
	Dyslipidemia: *N*	10	6	0.25
	Tobacco: pack years	5 ± 2 (0–55)	7 ± 2 (0–30)	0.51
	BMI: kg/m^2^	30 ± 7 (21–50)	24 ± 5 (9–31)	0.0003

Diabetes	Type of diabetes: 1/2	3/20	—	—
	Duration of diabetes: years	4.8 ± 4.1 (0–13)	—	—
	Insulin: yes/no	9/13	—	—
	Oral antidiabetic drugs: yes/no	17/5	—	—
	HbA_1_c: %	8.5 ± 2.9 (5.9–17)	—	—
	Diabetic nephropathy: yes/no	2/20	—	—
	MDRD: mL/min/1.73 m^2^	106 ± 40 (44–185)	—	—
	Diabetic neuropathy: yes/no	0/22	—	—

**Table 2:** Quantitative parafoveal parameter measurements and comparison between diabetic subjects and controls.

Imaging tool	Variable	Diabetic subjects		Controls		Difference^∗^	*P* ^∗∗^	CI 95%
		Mean ± SD	Range	Mean ± SD	Range	Mean ± SD		
OCTA	sFAZ (mm^2^)	0.322 ± 0.13	0.611–0.141	0.285 ± 0.15	0.086–0.771	0.037 ± 0.22	0.31	(−0.04; 0.11)
	dFAZ (mm^2^)	0.444 ± 0.15	0.209–0.716	0.398 ± 0.14	0.173–0.736	0.046 ± 0.22	0.20	(−0.03; 0.11)
	sDensity 1 (%)	49.05 ± 2.44	44.17–54.21	49.45 ± 4.83	42.22–66.54	0.39 ± 6.38	0.74	(−2.78; 1.99)
	dDensity 1 (%)	55.94 ± 5.48	47.63–70.72	55.46 ± 3.89	48.14–67.47	0.49 ± 7.80	0.74	(−2.4; 3.4)
	sDensity 2 (%)	54.50 ± 1.87	50.17–57.50	55.45 ± 2.14	50.48–59.08	0.96 ± 2.83	0.07	(−1.99; 0.08)
	dDensity 2 (%)	60.14 ± 2.01	55.99–64.64	60.65 ± 2.49	54.93–65.42	0.51 ± 3.05	0.36	(−1.63; 0.61)
	sPIA (mm^2^)	0.499 ± 0.08	0.331–0.618	0.513 ± 0.11	0.177–0.679	0.01 ± 0.15	0.62	(−0.07; 0.04)
	dPIA (mm^2^)	0.382 ± 0.09	0.202–0.549	0.403 ± 0.07	0.202–0.549	0.02 ± 0.12	0.38	(−0.07; 0.03)
	C: coefficient of enlargement of FAZ	0.43 ± 0.32	0.01–1.56	0.60 ± 0.61	−0.14–2.62	0.17 ± 0.7	0.14	(−0.39; 0.06)

SD-OCT	Retinal foveal thickness (*μ*m)	257 ± 34	160–314	277 ± 44	219–326	30 ± 97	0.003	(−34; −7)
	Choroidal subfoveal thickness (*μ*m)	264 ± 64	95–419	294 ± 68	144–484	21 ± 44	0.06	(−60; −1)

^∗^Median value; *P*^∗∗^: *P* value of the comparison with a Student's *t*-test of FAZ area between ND and DNDR groups; AO: adaptive optics; cmOCT: correlation mapping OCT; DNDR: diabetic patient with no diabetic retinopathy; sFAZ: foveal avascular zone area of the superficial capillary plexus; dFAZ: foveal avascular zone area of the deep capillary plexus; FFA: fundus fluorescein angiography; ND: nondiabetic patient; NS: nonsignificant; OMAG: optical microangiography; OCTA: OCT-angiography; PV-OCT: phase variance-OCT; SLO: scanning laser ophthalmoscopy; SS-OCT: swept source OCT.

**Table 3:** Foveal avascular zone area measurements in healthy subjects and diabetic patients without diabetic retinopathy in the literature.

Author	Method	Status	*N*	sFAZ	dFAZ	*P* ^∗∗^ (sFAZ, dFAZ)
				Mean ± SD (mm^2^)		
Tam et al. [42]	AOSLO	ND	10	0.323 ± 0.107	—	—
Chui et al. [45]	AOSLO	ND	32	0.32 ± 0.16	—	—
Popovic et al. [46]	Dual conjugate AO	ND	5	0.302 ± 0.100	—	—
Nelson et al. [47]	Retinal function imager	ND	37	0.125 ± 0.070	—	—
Conrath et al. [18]	FFA	ND	31	0.152 ± 0.086	—	—
John et al. [48]	FFA	ND	31	0.275 ± 0.074	—	—
Bresnick et al. [20]	FFA	ND	20	0.35^∗^	—	—
Mansour et al. [31]	FFA	ND	27	0.405 ± 0.559	—	—
Dubis et al. [43]	SLO-FFA	ND	42	0.420 ± 0.250	—	—
Arend et al. [49]	SLO-FFA	ND	52	0.205 ± 0.062	—	—
Kuehlewein et al. [50]	SS-OCT-OMAG	ND	19	0.304 ± 0.132	0.486 ± 0.162	—
Enfield et al. [51]	cmOCT	ND	37	0.125 ± 0.070	—	—
Kim et al. [30]	PV-OCT	ND	2	0.167	—	—
Shahlaee et al. [32]	OCTA	ND	44	0.27 ± 0.101	0.34 ± 0.116	—
Carpineto et al. [44]	OCTA	ND	6	0.251 ± 0.096	—	—
Samara et al. [38]	OCTA	ND	67	0.266 ± 0.097	0.495 ± 0.227	—
Arend et al. [24]	SLO-FFA	ND	21	0.231 ± 0.06	—	—
		DNDR	7	0.276 ± 0.08	—	NS
de Carlo et al. [16]	OCTA	ND	28	0.288 ± 0.136	—	
		DNDR	61	0.348 ± 0.101	—	0.04
Takase et al. [23]	OCTA	ND	19	0.25 ± 0.06	0.38 ± 0.11	<0.01 <0.01
		DNDR	24	0.37 ± 0.07	0.54 ± 0.13	
Di et al. [15]	OCTA	ND	85	0.36 ± 0.11		
		DNDR	53	0.40 ± 0.16		0.04
Present study	OCTA	ND	23	0.294 ± 0.150	0.348 ± 0.02	0.31 0.20
		DNDR	23	0.321 ± 0.120	0.420 ± 0.02	

^∗^Mean difference between cases' and controls' values; *P*^∗∗^: *P* values in the comparison between diabetic subjects and their age- and gender-matched nondiabetic controls with a paired Student's *t*-test; CI 95%: 95% confidence interval; sDensity1 and dDensity1: parafoveal ring vascular density measured with the “flow” quantification tool (method 1) respectively, on the SCP and DCP; sDensity2 and dDensity2: parafoveal ring vascular density measured with the “density” quantification tool (method 2) respectively, on the SCP and DCP; DCP: deep capillary plexus; FAZ: foveal avascular zone; sFAZ and dFAZ: FAZ area, respectively, on the SCP and DCP; sPIA and dPIA: parafoveal intercapillary area, respectively, on the SCP and DCP; SCP: superficial capillary plexus; SD: standard deviation; SD-OCT: spectral domain-OCT.

## Abbreviations

AOSLO: Adaptive optics scanning laser ophthalmoscopy
BMI: Body mass index
CI: Confidence interval
cmOCT: Correlation mapping OCT
DCP: Deep capillary plexus
sDensity1 and dDensity1: Parafoveal ring vascular density measured with the “flow” quantification tool (method 1), respectively, on the superficial and the deep capillary plexus
sDensity2 and dDensity2: Parafoveal ring vascular density measured with the automatized “density” quantification tool (method 2), respectively, on the superficial and the deep capillary plexus
DNDR: Diabetic patients with no diabetic retinopathy
DR: Diabetic retinopathy
EDI: Enhanced depth imaging
ETDRS: Early treatment diabetic retinopathy study
FAZ: Foveal avascular zone
sFAZ: FAZ area of the superficial capillary plexus
dFAZ: FAZ area of the deep capillary plexus
FFA: Fundus fluorescence angiography
HbA_1_c: Glycated hemoglobin
MDRD: Glomerular filtration rate calculated with the modification of diet in renal disease study equation
ND: Nondiabetic patients
NS: Nonsignificant
OMAG: Optical microangiography
OCT: Optical coherence tomography
OCTA: OCT angiography
PIA: Parafoveal intercapillary area
sPIA and dPIA: Parafoveal intercapillary area, respectively, on the superficial and the deep capillary plexus
PV- OCT: Phase variance-OCT
SCP: Superficial capillary plexus
SD: Standard deviation
SD-OCT: Spectral domain OCT
SLO: Scanning laser ophthalmoscopy
SS-OCT: Swept source-OCT
SSADA: Split-spectrum amplitude decorrelation angiography
AS: Anne Sikorav
AM: Alexandra Miere
MMG: Mathilde M. Goudot.
